# Molecular and Genetic Characterization of Natural HIV-1 Tat Exon-1 Variants from North India and Their Functional Implications

**DOI:** 10.1371/journal.pone.0085452

**Published:** 2014-01-23

**Authors:** Larance Ronsard, Sneh Lata, Jyotsna Singh, Vishnampettai G. Ramachandran, Shukla Das, Akhil C. Banerjea

**Affiliations:** 1 Virology Laboratory, National Institute of Immunology, New Delhi, India; 2 Department of Microbiology, University College of Medical Sciences and Guru Teg Bahadur Hospital, Delhi, India; University of Nebraska Medical Center, United States of America

## Abstract

**Background:**

Designing an ideal vaccine against HIV-1 has been difficult due to enormous genetic variability as a result of high replication rate and lack of proofreading activity of reverse transcriptase leading to emergence of genetic variants and recombinants. Tat transactivates HIV-1 LTR, resulting in a remarkable increase in viral gene expression, and plays a vital role in pathogenesis. The aim of this study was to characterize the genetic variations of Tat exon-1 from HIV-1 infected patients from North India.

**Methods:**

Genomic DNA was isolated from PBMCs and Tat exon-1 was PCR amplified with specific primers followed by cloning, sequencing and sequence analyses using bioinformatic tools for predicting HIV-1 subtypes, recombination events, conservation of domains and phosphorylation sites, and LTR transactivation by luciferase assay.

**Results:**

Phylogenetic analysis of Tat exon-1 variants (n = 120) revealed sequence similarity with South African Tat C sequences and distinct geographical relationships were observed for B/C recombinants. Bootscan analysis of our variants showed 90% homology to Tat C and 10% to B/C recombinants with a precise breakpoint. Natural substitutions were observed with high allelic frequencies which may be beneficial for virus. High amino acid conservation was observed in Tat among Anti Retroviral Therapy (ART) recipients. Barring few changes, most of the functional domains, predicted motifs and phosphorylation sites were well conserved in most of Tat variants. dN/dS analysis revealed purifying selection, implying the importance of functional conservation of Tat exon-1. Our Indian Tat C variants and B/C recombinants showed differential LTR transactivation.

**Conclusions:**

The possible role of Tat exon-1 variants in shaping the current HIV-1 epidemic in North India was highlighted. Natural substitutions across conserved functional domains were observed and provided evidence for the emergence of B/C recombinants within the ORF of Tat exon-1. These events are likely to have implications for viral pathogenesis and vaccine formulations.

## Introduction

Acquired immunodeficiency syndrome (AIDS) is a relentless pandemic disease among infectious diseases. The first AIDS case was detected among Indian sex workers in 1987. It has since spread to almost all the states of India due to socio-epidemiological reasons, the high genetic variability of human immunodeficiency virus (HIV) and high error rate of reverse transcription [1]. Increased virus production [2] and fast replication kinetics [3] leads to the generation of highly divergent and circulating recombinant strains. HIV-1 is classified into groups, subtypes, sub-subtypes, circulating recombinant forms (CRFs) and unique recombinant forms (URFs). The major groups are M, N, O and P, among which the P group was identified from Cameroon people [4]. The M group is the major group responsible for the AIDS pandemic and prevalent all over the world and it is divided into nine subtypes (A to D, F to H, J and K) and sub-subtypes (A1 and A2, F1 and F2) [5]. The O, N and P groups are prevalent in Central Africa and Cameroon. Subtype B is more prevalent in Western and Central Europe, North America and Australia. Subtypes A and D are prevalent in East and West Africa, and Russia. Subtype C is more prevalent in South Africa and South East Asia [6]. The generation of multiple subtypes are mainly due to the error prone mechanism of reverse transcription. During this process, viral reverse transcriptase (RT) can switch with high frequency between two templates of genomic RNA dimer by forced copy-choice model [7] and strand displacement-assimilation model [8] that results in the generation of highly divergent circulating recombinants. Recent studies reveal the increasing numbers of URFs and 55 CRFs have been characterized worldwide [9], testifying to the genetic diversity of HIV-1 in different geographical regions.

Epidemiological studies on subtype prevalence based on many HIV-1 strains isolated around the world suggest that HIV-1 is rapidly evolving with the generation of large numbers of quasispecies within infected patients during natural infection [10]. This is influenced by host immune responses, antiretroviral restriction factors and other selection mechanisms compelling the virus to evolve with optimum replication and adaptation efficiency [11]. The high genetic diversity among quasispecies has augmented the burden of HIV-1 infection, which is a major concern for developing countries like India. Genetic analyses of HIV-1 strains from different parts of India in the last 25 years showed the high prevalence of subtype C along with the emergence of certain recombinants such as A/C, A/E and B/C [12], which were based on sequence analyses of env, gag and pol genes, but no genetic information is available for tat exon-1 gene from North India. HIV-1 genome has a set of accessory genes (vif, vpr, vpu and nef), two regulatory genes (tat and rev) and three structural genes (gag, pol and env) [13]. Among the regulatory genes, Tat is known to play a vital role in HIV-1 lifecycle. It is produced early after infection and it strongly activates viral gene expression [14] from long terminal repeat (LTR) promoter through interaction with the transactivation response RNA (TAR) element [15] resulting in a drastic increase of viral transcription [16]. It was reported earlier that natural variation in Tat had differential impact on LTR driven transcription and apoptosis [17]. Since Tat plays an important role in HIV-1 pathogenesis, it is important to genetically characterize Tat exon-1 in our population.

Genetic analysis of Tat exon-1 variants from HIV-1 infected patients from North India showed the predominance of subtype C along with emerging B/C recombinants. The possible sequence similarity of our variants was determined using phylogenetic analysis by comparison with global reference sequences from different geographical regions. Except few variants, most of our variants showed high amino acid conservation at various functional domains with few natural substitutions which may be beneficial for the virus. Most of the functional domains, motifs and phosphorylation sites were well conserved. The dN/dS values were less than one for all our variants suggestive of purifying selection. Tat C variants (TatN12 and TatD60) and B/C recombinant (TatVT6) showed varying levels of LTR transactivation. We extensively discuss the possible role of Tat exon-1 variants in shaping North Indian HIV-1 epidemic and highlight the possible functional implications of this study for the development of preventive measures.

## Results

### Clinical data of HIV-1 infected patients

Blood samples of HIV-1 infected patients (n = 120) residing in Himachal Pradesh (n = 18), Punjab (n = 18), Haryana (n = 16), Chandigarh (n = 22), Uttar Pradesh (n = 20) and Delhi (n = 26) of North Indian regions were collected from 2004 to 2010. The requisite ethical clearances were obtained from the host institutes. HIV-1 infected patients included men (n = 68) and women (n = 52) with an average age of 32 years, mother to child infected pairs (n = 22), mothers with an average age of 33 years, children with an average age of eight years. 82% of samples were from heterosexual acquisitions and 18% of samples were from vertical transmission. CD4 count for heterosexuals ranged from 39 to 1046 cells/mm^3^ (mean = 250 cells/mm^3^); for infected children the counts ranged from 458 to 1048 cells/mm^3^ (mean = 764 cells/mm^3^) and for infected mothers the counts ranged from 96 to 811 cells/mm^3^ (mean = 306 cells/mm^3^). ART recipients (43%) and ART naive (57%) patient samples were included in this study. The clinical data of patients is given in Table 1 along with supporting information (Table S1).

**Table 1:** Clinical data of HIV-1 infected patients from North India during 2004–2010 (n = 120).

	Mean	Range	Percentage
**Age (yrs)**	31	4 to 54	-
**Sex (%)**	-	-	57% men and 43% women
**Route of transmission (%)**	-	-	82% heterosexual and 18% vertical transmission
**Positive since detection (yrs)**	6	2004 to 2010	-
**ART status (%)**	-	-	ART recipients (43%) and ART naive (57%)
**CD4 counts (cells/mm^3^)**	250 cells/mm^3^ for heterosexuals, 764 cells/mm^3^ for infected children and 306 cells/mm^3^ for infected mothers	39–1046 cells/mm^3^ for heterosexuals, 458–1048 cells/mm^3^ for infected children and 96–811 cells/mm^3^ for infected mothers	-

### PCR amplification and TA cloning

Genomic DNA was isolated from peripheral blood mononuclear cells (PBMCs) of HIV-1 infected patients by QIAamp DNA Blood Mini Kit. Tat exon-1 gene was amplified from genomic DNA by polymerase chain reaction (PCR). Tat exon-1 amplicons of size 215 base pair (bp) were cloned into pGEM-T Easy vector, screened for positive clones by restriction digestion with *EcoRI* and positive clones were sequenced; three independent clones from each individual was sequenced to rule out laboratory contamination and were further cross checked by ClustalW 2.1. Cloning and sequencing were carried out twice for the positive clones to avoid PCR generated errors. A known HIV-1 NL4-3 sequence was included for amplification as a control to assess errors generated by Takara Taq polymerase.

### Phylogeny based HIV-1 sub-typing and sequence similarity

**Figure 1:**
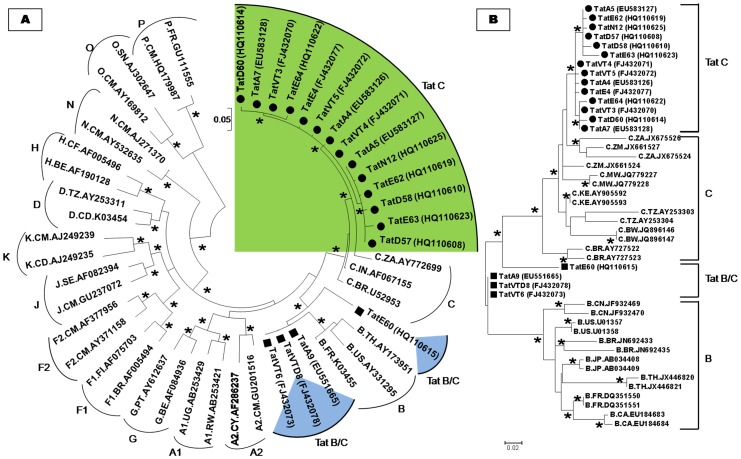
HIV-1 sub-typing based on global subtype references. **A**) The phylogenetic tree of Tat exon-1 variants with M (A to K including A1, A2, F1, and F2), N, O and P groups. **B**) The phylogenetic tree of Tat exon-1 variants with global subtype B from countries such as China (CN), France (FR), Japan (JP), Thailand (TH), United States (US), Canada (CA) and Brazil (BR) and global subtype C from countries such as Botswana (BW), Tanzania (TZ), South Africa (ZA), Zambia (ZM), Kenya (KE), Malawi (MW) and Brazil (BR) sequences. Each reference sequence was labelled with a subtype, followed by country of isolation and accession number. Filled circles represent our C variants and filled rectangles represent our B/C recombinants. The bootstrap probability (>65%, 1,000 replicates) was showed with asterisk (*) at the corresponding nodes of the tree and the scale bar represents the selection distance of 0.05 and 0.02 nucleotides per position in the sequence.

The nucleotide sequences of Tat exon-1 were aligned with ClustalW 2.1 [18] and phylogenetic analysis was carried out using the neighbour joining method [19] with Kimura two parameter distance matrix in MEGA5 software [20] with M group subtypes (A–K) and sub-subtypes (A1, A2, F1 and F2). This analysis showed that 90% of variants clustered with Tat C and 10% of variants branched between Tat B and C showing the occurrence of B/C recombination events (Figure 1A). To identify the possible sequence similarity of our variants, phylogenetic tree was constructed using maximum likelihood method with a collection of B and C references from different parts of the world. This analysis revealed that our C variants were close to Tat C from South Africa, Zambia and Malawi pointing out to its South African origin. B/C recombinants showed sequence similarity to subtype B from America, France, China, Thailand and subtype C from South Africa, Zambia, Malawi showing complex origin and multiple introductions of B and C subtypes from different population (Figure 1B).

### Recombination events and nucleotide breakpoints

**Figure 2:**
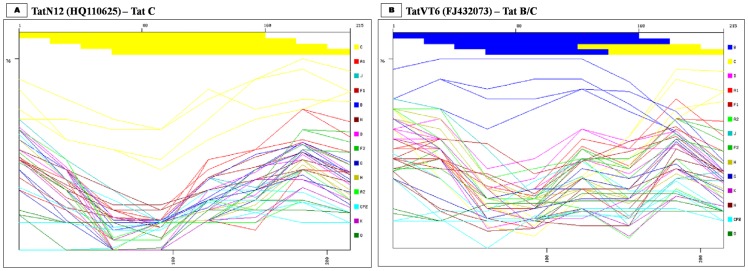
Recombination events identification. **A**) TatN12 is a representative of Tat exon-1 C variant. **B**) TatVT6 is a representative of Tat exon-1 B/C recombinant. Reference subtypes (A1, A2, B, C, D, F1, F2, G, H, J, K, N, O and CPZ) were used to genotype our variants using retroviruses genotype tool from NCBI. The yellow line represents subtype C and the blue line represents subtype B. The X-axis represents the percentage of sequence similarity to the corresponding subtypes and the Y-axis represents the amino acid position of variant sequence.

**Figure 3:**
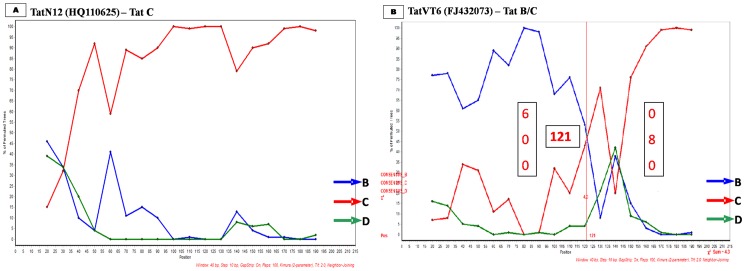
Breakpoints prediction at specific nucleotides. **A**) TatN12 is a representative of Tat exon-1 C variant. **B**) TatVT6 is a representative of Tat exon-1 B/C recombinant. Bootscan analysis was performed using consensus B, C and D sequences as shown in blue, red and green arrows respectively with our variants. The X-axis represents the percentage of sequence similarity with the corresponding subtypes and the Y-axis represents the amino acid position of variant sequence. Informative site analysis of B/C recombinants suggested that the N-terminal half consisted of 6, 0 and 0 values for subtypes B, C and D respectively and the C-terminal half consisted of 0, 8 and 0 values for Tat B, C and D respectively.

The possible recombination events in our variants were predicted using NCBI retroviruses genotype tool [21] using 80 bp window size/20 bp step size. Our sequences were compared with A1, A2, B, C, D, F1, F2, G, H, J, K, N, O and CPZ sequences. This analysis revealed that 90% of our variants aligned with subtype C (Figure 2A) and 10% variants aligned with N-terminal of subtype B and C-terminal of subtype C, indicating the presence of B/C recombinants (Figure 2B). Bootscan analysis was performed with consensus Tat B, C and D sequences using 40 bp window size/10 bp step size with kimura two parameter in Simplot 3.5.1 [22] which confirmed the earlier sub-typing results that 90% variants was close to subtype C (Figure 3A) and 10% variants was B/C recombinants with an unique breakpoint at 144^th^ nucleotide position (Figure 3B). In addition, amino acid sequence alignment of Tat exon-1 variants with consensus B and C sequences showed similarity to Tat B in the N-terminal, cysteine rich, core and arginine rich regions, but the glutamine rich region showed similarity to Tat C (data not shown).

### Mutational analysis of Tat exon-1 variants

The nucleotide sequences of Tat exon-1 variants were translated into amino acid sequences and aligned with consensus Tat B and C. Despite high amino acid conservation, some interesting mutations were observed in various functional domains which included K24T, H29Y and L35P mutations in cysteine-rich region with 1.000, 1.000 and 0.333 allelic frequencies respectively; G44S and S46F mutations in the core region with 30% and 20% of variants respectively; P68L mutation in all variants in the glutamine rich region which was neither seen with Tat B nor Tat C consensus sequences. Additional mutants like F38L, Q39M, K41N and G42A were found with 10%, 8%, 6% and 6% frequency respectively. Taken together, seven mutations (K24T, H29Y, L35P, G44S, S46F, P60Q and P68L) were highly prevalent. Four mutations (F38L, Q39M, K41N and G42A) were observed with less than 0.200 allelic frequencies (Table 2).

**Table 2:** Mutations in Tat exon-1 variants.

Novel mutation	Functional domain	Allele frequency
K24T	Cysteine rich	1.000
H29Y	Cysteine rich	1.000
L35P	Cysteine rich	0.333
F38L	Core region	0.100
Q39M	Core region	0.083
K41N	Core region	0.066
G42A	Core region	0.066
G44S	Core region	0.300
S46F	Core region	0.200
P60Q	Arginine rich	1.000
P68L	Glutamine rich	1.000

Mutational study on the residues that are involved in post translational modifications of Tat [23] is important to determine the functional activity of Tat. The basic domain of Tat had the six arginine (R) residues in Tat C and seven R residues in Tat B. These are essential substrates for arginine methylation [24]. Indian Tat variants showed six R residues which were similar to Tat C, but 10% variants showed five R residues due to substitution of R with W at 52^nd^ position, and may influence LTR transactivation. The cysteine rich region of Tat had seven cysteine (C) residues in Tat B and six C residues in Tat C and are essential for CCR5 expression in monocytes [25]. 90% variants showed six cysteine and 10% variants showed seven cysteine residues which were subtype specific variation; any change in these residues are known to modulate CCR5 expression. The core domain of Tat contributes to Tat-TAR RNA interaction [26]. Except amino acid change at 35^th^ position with glutamine (Q) in 30% variants, the core region remained conserved in our variants. The arginine-rich region of Tat had six and five R residues in Tat B and C respectively which are required for TAR binding and any change in these residues can alter transactivation potential [27]. All our Tat variants showed five R residues. The lysine (K) residues at 28, 50, 51 and 71^st^ positions were highly conserved in our variants which are essential for binding with p300/CBP-associated factor (PCAF) to activate the viral transcription by methylation [28]. Any alteration in these residues can potentially change the Tat-TAR interaction.

The glutamine rich region of Tat had four Q residues at 55, 60, 63 and 66^th^ positions in Tat B whereas Tat C had two Q at 55 and 66^th^ positions, and are essential for transactivation through TAR interaction [29] and these residues had also been reported to be involved in T cell apoptosis [30]. Interestingly, our variants despite being subtype C possessed three Q at 55, 60 and 66^th^ positions instead of two Q residues. The N-terminal acidic domain of Tat (negatively charged amino acids) was predicted to form an alpha helix. Beside, these negatively charged amino acids, Tat B had two positively charged residues (R7 and K12) that are most probably involved in stabilizing the secondary structure of Tat protein, but Tat C lacked those residues; R7 and K12 were substituted with asparagine (N) in 90% of our variants which can change the net charge of Tat protein and may hinder alpha helix formation. In the case of Tat B/C recombinants, amino acid analysis revealed sequence similarity pattern with Tat B at various amino acid positions (K12, T23, N24, K28, C30, F31, Q34, M39, A42 and Q60).

**Figure 4:**
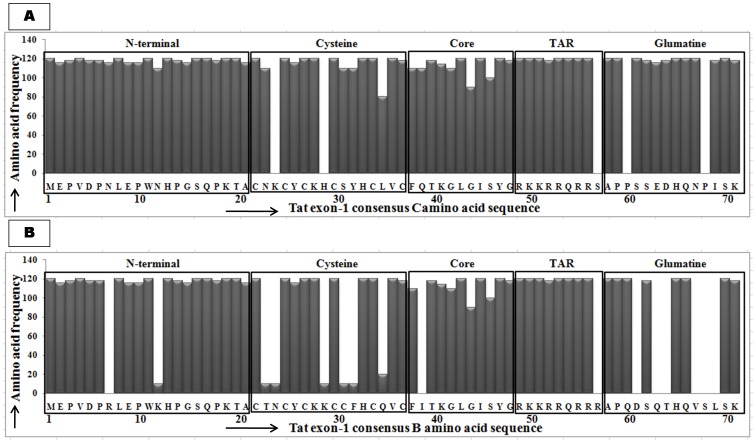
Tat exon-1 amino acid sequence analysis. **A**) Amino acid signature pattern of Tat exon-1 variants were compared with consensus Tat B sequence. **B**) Amino acid signature pattern of Tat exon-1 variants were compared with consensus Tat C sequence. The X-axis represents the amino acid consensus sequence of Tat B and C with the functional domains such as N-terminal (1–21aa), Cystine rich (22–37aa), Core (38–48aa), Arginine rich (49–57aa), Glutamine rich (58–71aa) and the Y-axis represents the amino acid frequency observed in our variants.

Despite these variations, alignment with Tat C showed amino acid conservation at the N-terminal acidic, core and arginine rich TAR regions (Figure 4A); notably substitutions such as K24T, H29Y, L35P and P68L were observed in our variants that were not seen with consensus Tat C. On the other hand, alignment with Tat B showed amino acid conservation at TAR region, but other regions showed specific variations (Figure 4B); remarkably 16 conserved substitutions were observed in our variants that were not seen with consensus Tat B.

### Motifs and phosphorylation sites in Tat exon-1 variants

**Figure 5:**
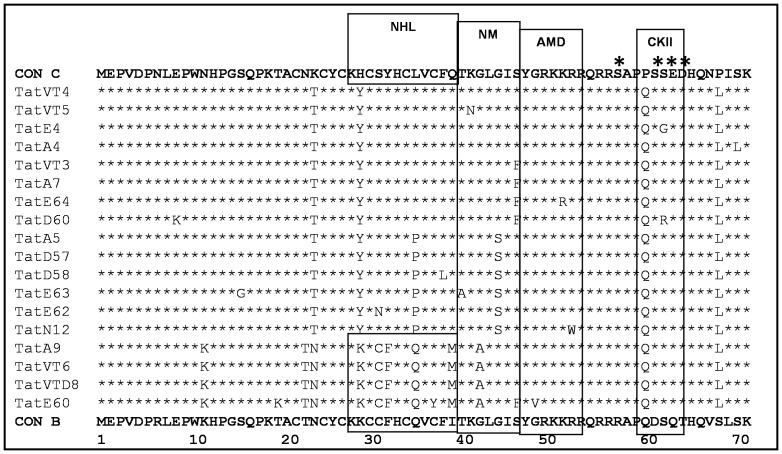
Motifs and phosphorylation sites analyses. Motifs and phosphorylation sites were predicted for Tat exon-1 variants using Motif scan and NetPhos 2.0 program respectively by comparing with Tat B and C consensus sequences. Tat exon-1 variants show NCL-1_HT2A_Lin-41 homologous protein (NHL) site (29–41aa), N-myristoylation (NM) site (42–47aa), amidation (AMD) site (47–50aa) and casein kinase II (CKII) site (61–64aa). The phosphorylation sites (serine and threonine residues) were marked with asterisk (*) at the corresponding amino acid sites on the top of the consensus C sequence.

Interferon (IFN) induces dsRNA dependent serine/threonine protein kinase (PKR) which exerts IFN-mediated antiviral activity by blocking translation in infected cells through phosphorylation of the alpha subunit of elongation factor 2 (eIF2). Tat acts as a substrate homologue for the enzyme which in turn competes with eIF2α and inhibits the translation. It had been shown that during the interaction with PKR, Tat is phosphorylated at three residues; S62, T64 and S68 [31], which helps in increasing the interaction with TAR and enhances viral transcription [32]. S62 was conserved in our variants, but S68 and T64 were substituted with leucine (L) and aspartic acid (D) respectively in all Tat variants. Phosphorylation site analysis using NetPhos 2.0 [33] showed that S57 and S61 sites were well conserved in our variants (shown with asterisk in Figure 5). Motif analysis using Motifscan [34] showed casein kinase II (CKII), amidation (AMD) and N-myristoylation (NM) motifs that were conserved in all our variants (Figure 5). Surprisingly, the predicted NCL-1_HT2A_Lin-41 homologous protein (NHL) motif was not detected in Tat B and C variants due to substitution of histidine (H) at the 30^th^ position with tyrosine (Y) but Tat B/C recombinants retained this motif, which is known to play an important role in bridging integrin to the cytoskeleton and involved in protein-protein interactions [35]. The exact role of NHL motif in HIV-1 infection is not known at present and warrants further study.

### Selection pressure based on dN/dS ratio

To find the type of selection that occurred with Tat exon-1 variants, the dN/dS values within the predicted subtypes of our variants were calculated using SNAP 1.1.0 (Synonymous Non-synonymous Analysis Program) tool. SNAP calculates non-synonymous (dN) and synonymous (dS) substitution rates based on a set of codon-aligned nucleotide sequences (dN/dS ratio) [36]. The dN/dS value for Tat exon-1 variants ranged from 0.33 to 0.51. All our Tat exon-1 variants including B/C recombinants showed dN/dS values less than one which indicates purifying selection. Further, the type of selection that occurred with B and C variants were calculated by determining the average divergence among B and C subtypes which also showed dN/dS values less than one which again confirmed the purifying selection (Table 3).

**Table 3:** dN/dS ratio for unique Tat exon-1 variants.

Sample	dN/dS ratio (Consensus C)	dN/dS ratio (Consensus B)	Predicted subtype	Selection type
TatN12	0.3034	0.4030	C	Purification
TatD60	0.3266	0.4515	C	Purification
TatVT5	0.3170	0.4432	C	Purification
TatVT4	0.3560	0.4772	C	Purification
TatE63	0.3680	0.5110	C	Purification
TatVT6	0.4820	0.3674	B/C	Purification
TatVTD8	0.4290	0.3345	B/C	Purification
TatE60	0.4354	0.3520	B/C	Purification

### Transcriptional levels of Tat C variants and B/C Tat

**Figure 6:**
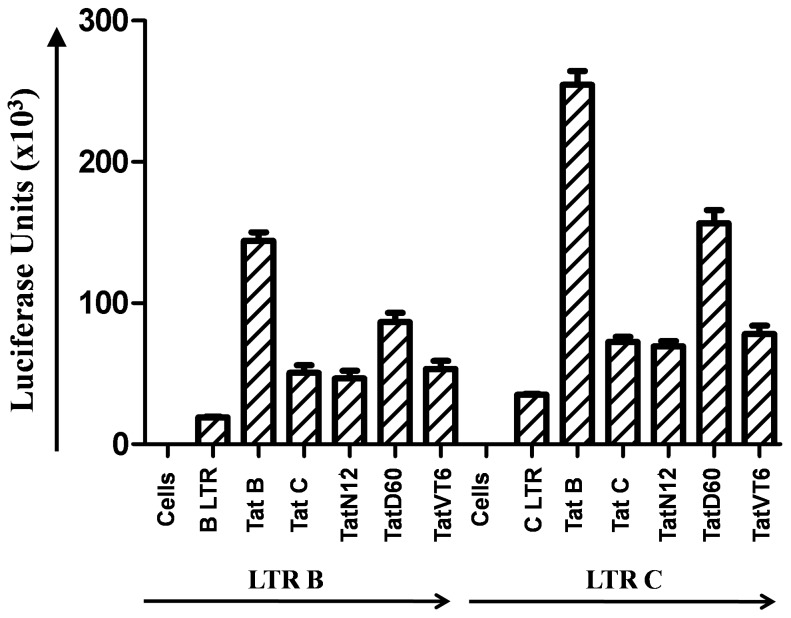
HIV-1 LTR Transactivation by Tat C variants and B/C Tat. HEK293 cells were co-transfected with 200 ng Tat variants (TatN12, TatD60 and TatVT6) and 50 ng of pGL3-Luc vector containing subtype B or C LTR, and pGL3-Luc B or C LTR alone was used as control. After 24 hours of transfection, cells were lysed with lysis buffer and the luciferase activity was measured in a luminometer. The relative transactivation was expressed as mean luciferase units obtained from luciferase assay. The error bar represents the standard deviation of luciferase units and the p values less than 0.05 represents the significance of luciferase activity.

To determine the impact of Tat variants on HIV-1 LTR transactivation, in vitro studies were carried out in HEK293 cell line with selected unique Tat C variants (TatN12, TatD60) and B/C Tat (TatVT6) along with prototype Tat B and C. To delineate the complexity of Tat functional domains, B and C LTR luciferase reporter gene constructs were co-transfected along with Tat variants and the luciferase units were measured. Tat B showed more significant (p<0.05) in causing LTR transactivation than Tat C variants. TatN12, a subtype Tat C showed transactivation at a level similar to Tat C. TatD60, a subtype Tat C showed a significantly higher (p<0.05) LTR transactivation than Tat C. TatVT6, a B/C Tat showed little higher transactivation than Tat C but the increase was less significant (Figure 6). The same experiment was also tested in other cell lines like MCF-7 cell line [37] (data not shown) and similar levels of transactivation was observed with Tat exon-1 variants.

## Discussion

Genetic analyses of HIV-1 variants from South and North east region of India was carried out earlier which showed high prevalence of subtype C with circulating recombinants in different states of India [38]–[40]. HIV-1 exhibits distinct distribution of genotypes in different geographical niche with varying clinical outcomes, prognosis and responses to ART [41]. Recent studies suggest that evolving recombinants are more potent in causing enhanced pathogenesis via transactivation, replication and other possible mechanisms [42]. CRFs arise due to co-circulation of many viral genotypes during natural infection [43]. We have earlier characterized some HIV-1 genes which included vpu, vpr, env and rev that revealed the co-circulation of B and C subtypes in North India [44]–[46]. Limited information is available about a Tat gene from North East India among Injecting drug users (IDUs) [47] but no data are reported from heterosexuals residing in North India.

Since first exon of Tat has major functional domains and is sufficient for HIV-1 LTR transactivation, we carried out extensive genetic analysis of the first exon of Tat gene. Genetic analysis revealed the presence of subtype C and B/C recombinants in the open reading frame (ORF) of Tat exon-1. This proportion of B/C recombinants were statistically significant (p<0.05). This would mean that the proportion of recombinants have increased marginally (∼3 to 5%) but steadily over the last decade enlisting a stable evolution of the virus in this region of our country. B/C recombinants have a common breakpoint which suggests that this region of Tat may be the hotspot for the generation of inter-subtype recombinants. Heterosexuals are vulnerable to HIV-1 infection due to unprotected sex with multiple sexual partners. They act like a bridge in spreading infection from high risk groups to healthy people. This study establishes the close relationship among Tat B/C variants with the variants from heterosexuals of China and neighbouring countries of India. Our multiple natural subtype C variants showed sequence similarity with South African variants. B/C recombinants showed sequence similarity to variants from different geographic regions which suggest multiple introductions of strains from China and other countries such as Thailand, France and America. Increased global travel may account for this observation.

The natural substitutions in Tat gene was earlier reported to increase viral gene expression [17]. Our mutational analysis suggests that certain conserved natural substitutions may be associated with increased viral fitness, but the exact mechanism and the role of these mutations can be known by carrying out extensive functional studies of these variants. It is important to note that the target residues for methylation, acetylation, phosphorylation and ubiquitination of Tat were well conserved suggesting their functional importance. Motif site analysis showed conservation of various motifs across the functional domains. Phosphorylation site analysis showed five important sites in Tat exon-1 variants, out of which three were well conserved (S62, S57 and S61) and the other two (S68 and T64) sites were substituted with L and D respectively in all our variants. These substitutions may cause drastic changes in various functions of Tat. The dN/dS analysis suggested that our variants were under purifying selection and that only limited sequence variations are permitted to preserve the functional activity of Tat. No significant genetic diversity was observed among HIV-1 infected children in comparison with infected mothers, but interestingly we observed low viral loads with improved CD4 counts in infected children receiving ART than their mothers demonstrating the clinical importance of ART.

Since subtype-specific differential activity had already been an acknowledged aspect of HIV-1 pathogenesis [48], inter-subtype recombinants may influence AIDS progression. Earlier we reported the differential expression of various viral proteins of subtypes B and C with respect to their functions [49], [50] but viral or cellular components responsible for these differences have not been studied in detail and in most cases the molecular determinants are not known. Tat had already been known to display subtype-specific differential activity during HIV-1 infection. Studies based on Tat from different geographic locations will obviously have major implications for overall viral pathogenesis.

It was reported that Tat interacts with TAR and is involved in transactivation of LTR resulting in high viral gene expression [51]. The acidic N-terminus region of Tat has thirteen amphipathic amino acids which are important for transactivation [52]. The cysteine region has seven cysteine residues which are highly conserved, while any change in these residues abolishes Tat function [53]. The core region is close to the proximity of Tat-TAR bulge region, which is important for stability of Tat-TAR interaction [54]. The arginine region has six arginine residues and the glutamine region has three glutamine residues which are essential for TAR binding [52]. Tat E had the high transactivation capacity than Tat B and Tat C [55], and CRF12_BF transactivates higher than Tat B [56] which shows the subtype specific Tat activity on LTR transactivation. Several substitutions in Tat were reported to play crucial role in transactivation and these include K28, K41, K50, K51, K71 which are essential for acetylation by p300/PCAF, R57 and R56 and are required for TAR interaction. Y 47, C22, C31 and C34 are required for LTR transactivation [57]. Tat C variants (TatN12 and TatD60) and B/C recombinant (TatVT6) showed differential LTR transactivation. This differential activity was due to genetic variations in Transactivation and TAR domains. TatD60, a subtype Tat C showed high transactivation than Tat C which could be due to unique mutation (S46F). TatVT6, a B/C Tat showed increased transactivation which may be due to subtype C specific changes in the N-terminus and subtype B specific changes in the C-terminus.

For the last two decades, most of the HIV-1 vaccines are mainly focused on env, gag, and pol genes. However these approaches have failed to induce protection against HIV-1 in clinical trials due to high rate of mutations rendering elimination of heterologous viruses difficult [58]. For instance, phase III clinical trials based on env from VaxGen was not successful [59]. Similarly, other approaches based on gag, pol and nef genes had been developed by Merck for induction of T-cell mediated responses against HIV-1 which also failed [60]. All these failures have prompted us to pursue a different approach focusing on Tat, a major regulatory protein and is relatively conserved among different HIV-1 subtypes [61]. Tat is produced early after infection and is essential for virus replication and infectivity [62]. In the absence of Tat protein, trace amounts of structural proteins are produced and therefore, no infectious virus is formed [63]. During acute infection, Tat is taken up by monocyte derived dendritic cells (MDDC) and promotes MDDC maturation and activation resulting in maximum antigen presentation, leading to increased T cell responses against heterologous antigens [64]. CD8+ T cell-mediated responses (CTL) are also induced through major histocompatibility complex (MHC) class I pathway [65], [66]. Mainly, Tat transactivates viral gene expression and replication within the cells and favours the transmission of macrophage-tropic and T lymphocyte-tropic HIV-1 strains through induction of CCR5 and CXCR4 co-receptors [67]. Tat is also involved in the pathogenesis of AIDS and associated malignancies (Kaposi's sarcoma) [68]. Tat possesses immune-dominant epitope [69] which has high antigenicity and adjuvant properties that are beneficial for vaccine development. Italian AIDS vaccine team has conducted pre-clinical studies based on Tat which was found to be safe and immunogenic in phase I clinical studies and associated with slower progression to AIDS [70], [71]. Altogether, these peculiar properties make Tat, a vital candidate for vaccine either alone or in combination with other antigens for preventive and therapeutic purposes.

No single vaccine is likely to have universal application because of genetic variations, co-receptor tropism and ethnic diversity. It becomes essential to have a matrix of variants in affected population. Analysis of Tat exon-1 variants in a specific regional population will have a predictive value in determining the type of Tat based vaccine that is likely to give optimum immune response and protection in that particular population. Molecular characterization of Tat exon-1 based on sequence analysis of 120 HIV-1 infected patients from North India showed high amino acid conservation, particularly the transactivation and the TAR binding domains; these vital domains are essential for viral replication and pathogenesis. Present study on Tat exon-1 resulted in the identification of common sequences such as TatN12, TatD60, TatVT6 and TatVT5. These variants can be used in combination for making Tat based vaccination in our population because these variants may elicit efficient neutralising antibodies, a major challenge in the development of vaccines against HIV.

## Conclusion

Growing numbers of circulating recombinants have increased globally in the last few years. This rapid increase is the major cause of concern for developing countries like India. Genetic analysis of Tat exon-1 revealed that our variants predominantly clustered with subtypes C followed by B/C recombinants. It is important to continually monitor the genetic changes that HIV-1 undergoes in this region. Phylogenetic analyses of these natural variants are an effective way to explore the relationship between our variants with the dominant strains from different geographic regions. The observed natural substitutions at various positions of Tat exon-1 may involve in post translational modifications and might modulate Tat functions, therefore targeting these substitutions could lead to altered viral pathogenesis. High amino acid conservation was observed at domains, motifs and phosphorylation sites of Tat which are important for various functions of Tat. Purifying selection was observed in Tat exon-1 variants in our population which will help in the development of Tat based vaccine. The genetic variations in the functional domains of Tat exon-1 variants and B/C recombinants resulted in high levels of LTR transactivation which will obviously enhance viral replication and pathogenesis. This study indicates the importance of ART treatment in rendering the ability of virus to generate heterologous strains among North Indian population. Continued molecular surveillance and genetic analyses of HIV-1 genes for detecting possible genetic changes and recombination events will help in understanding the dynamic spreading tendency of HIV-1 virus in our population. This study provides genetic information of Tat exon-1 that will help in developing effective immunogens against a wide range of circulating strains in our country.

## Methods

### Patient selection and ethics statement

HIV-1 infected patients (n = 120) were collected on a cross sectional basis from North India who were registered and monitored at the immunodeficiency clinics of Guru Teg Bahadur (GTB) hospital, Delhi and Post Graduate Institute of Medical Education and Research (PGIMER), Chandigarh, during the period from 2004 to 2010. Both men (57%) and women (43%) were chosen for this study along with vertical transmission variants (18%). This study was approved by research project advisory committee, Institutional bio-safety committee and Institutional ethical committee for human research of University College of Medical Sciences (UCMS) and GTB hospital, Delhi, India and from PGIMER, Chandigarh, India. These institutes are mentored by National AIDS Control Organization (NACO), Ministry of Health and Family welfare, Government of India that provides free ART to HIV-1 seropositive patients under a structured HIV/AIDS control program. These ethics committees approved the written informed consent which we obtained from HIV-1 infected patients and from the guardians of HIV-1 infected children participants involved in this study.

### DNA isolation and PCR

Genomic DNA was extracted from PBMCs of HIV-1 infected patients by QIAamp DNA Blood Mini Kit (Qiagen) and sequence spanning Tat exon-1 was amplified by PCR using the following primers:

Forward primer: 5′- ATGGAGCCAGTAGATCCTAACCTA-3′

Reverse primer: 5′- TTGCTTTGATATAAGATTTTGATGATCCT-3′

PCR was carried out in a 15 µL reaction volume. The reaction mixture contained 500 ng genomic DNA (2.0 µL), 10× PCR Buffer (1.5 µL), 10 mM dNTP mix (0.37 µL), 1 µL of each primer (25 pmol), 0.25 µL of Takara Taq DNA polymerase and 8.88 µL of DNase/RNase free water. PCR conditions for the above primer sets were as follow: Initial denaturation at 94°C for 5 minutes (1 cycle), 30 cycles of denaturation at 94°C for 15 seconds, annealing at 63°C for 30 seconds and extension at 72°C for 40 seconds, and a final extension at 72°C for 5 minutes (1 cycle). PCR amplified products were analyzed on 1.5% agarose gel.

### Cloning and sequencing

The gel purified PCR products were cloned in pGEM-T Easy vector (Promega). The ligation reaction was incubated at 4°C for 10 hours then the ligation mix was added to LB ampicillin plates with *E.coli* DH5α strain. The plates were incubated overnight at 37°C. The positive clones were selected by picking a single colony and grown in 5 ml LB Broth with ampicillin (100 µg/ml) and incubated overnight at 37°C. Plasmid DNA was isolated from the culture by QIAprep Spin Mini Kit (Qiagen). The positive clones were screened by restriction digestion of plasmid DNA with *EcoRI* in a 10 µL reaction volume at 37°C for 2 hours. The digested products were analyzed on a 1.5% agarose gel. The positive clones were sequenced from LabIndia and SciGenom laboratories.

### HIV-1 sub-tying and sequence similarity analyses

The nucleotide sequences were assembled and error was checked by using BLAST to search for sequence similarities to previously reported sequences in the databases and to eliminate potential laboratory errors. These sequences were aligned with consensus sequences of HIV-1 strains of all subtypes using the ClustalW 2.1 [72]. Phylogenetic analyses were performed using the neighbour joining method (sub-typing) and maximum likelihood method (sequence similarity) with Kimura two-parameter distance matrix in MEGA5. The reliability of the node was tested using the bootstrap method with 1000 replicates. The phylogenetic trees were constructed based on Tat exon-1 representative variants from North India and reference sequences which were retrieved from a HIV sequence database that included subtypes B and C sequences obtained from different parts of the world including America, Japan, France, China, Thailand, Brazil, Kenya, Botswana, Zambia, South Africa and other countries.

### Recombination events analysis and dN/dS ratio calculation

Sub-typing and recombination events were verified using the genotype tool for retroviruses in the NCBI [73] and the results were confirmed using REGA HIV-1 sub-typing tool 2.0 [74] and Recombinant Identification Program (RIP) in the HIV databases. The bootscan analysis was performed using consensus B, C and D with gene-specific window size and step size in Simplot 3.5.1 to predict the breakpoints within our variants. SNAP 1.1.0 [75] was used to determine the nucleotide sequence selection pressure in our variants.

### Mutations identification and amino acid conservation analyses

The nucleotide sequences of Tat exon-1 were translated into amino acid sequences by Gene Runner. The multiple sequence alignments were made for all variants with consensus B and C using ClustalW 2.1. The novel mutations were identified and allelic frequencies were calculated from total samples (n = 120). The extent of amino acid sequence conservation was determined from the multiple sequence alignments of our variants with the corresponding consensus B and C sequences using a sequence alignment tool in MEGA5.

### Motifs and phosphorylation sites analyses

The motifs and phosphorylation sites in our variants were analyzed using Motif scan [76] and NetPhos 2.0 [77] by comparing the consensus B and C sequences.

### Cell culture, transfection and plasmids

HEK-293T (Human Embryonic Kidney 293 cells; NIH AIDS Reagent Programme) and MCF-7 cells were maintained in Dulbecco's modified Eagle's medium (DMEM) with 10% fetal bovine serum, 100 units penicillin, 0.1 mg streptomycin and 0.25 µg amphotericin B per ml at 37°C in the presence of 5% CO2. All transfections were performed using lipofectamine 2000 (Invitrogen) reagent. pcDNA3.1 Tat B, pcDNA3.1 Tat C, pcDNA3.1 TatN12, pcDNA3.1 TatD60, pcDNA3.1 TatVT6, pGL3-Luc B LTR, and pGL3-Luc C LTR were used in the experiments.

### Luciferase reporter assay

HEK293 cells were co-transfected with 200 ng of pcDNA3.1 Tat B, pcDNA3.1 Tat C, pcDNA3.1 TatN12, pcDNA3.1 TatD60 and pcDNA3.1 TatVT6 in each well of 6 well plates along with 50 ng of pGL3-Luc vector containing B or C LTR. Cells were transfected only with B or C LTR construct was used as control. After 24 hours of transfection, cells were harvested and lysed with reporter lysis buffer (Promega) and luciferase activity was measured in luminometer. The representative results were determined from the mean of three independent experiments.

### Statistical analysis

Statistical calculations were analyzed with a chi-square test using Graph Pad Prism 5.00. P value<0.05 was considered to be significant.

### Accession numbers

[GenBank: FJ432068–FJ432079, FJ210870–FJ210875, EU583126–EU583128, EU551665, FJ429357, FJ429358, HQ110624–HQ110630, HQ110608–HQ110623, JQ918787–JQ918788, GU451679–GU451681, HQ011384–HQ011385].

## Supporting Information

Table S1 **Clinical data of HIV-1 infected patients (n = 120) collected from the immunodeficiency clinics of GTB hospital, Delhi and PGIMER, Chandigarh, India.**
(DOC)
