# Supplementary material for: Molecular and Genetic Characterization of Natural HIV-1 Tat Exon-1 Variants from North India and Their Functional Implications
Source: PLoS One. 2014 Jan 23;9(1):e85452. doi: 10.1371/journal.pone.0085452 (PMC3900424; doi:10.1371/journal.pone.0085452)
Supplement: Table S1 — Clinical data of HIV-1 infected patients (n = 120) collected from the immunodeficiency clinics of GTB hospital, Delhi and PGIMER, Chandigarh, India. (DOC) [file pone.0085452.s001.doc]

**Supporting Information:**

| **Table S1 Clinical data for HIV-1 infected patients from North India** | | | | | | | |
| --- | --- | --- | --- | --- | --- | --- | --- |
| Samples | Age (yrs) | Sex | Route of transmission | | Positive since detection | ART status | CD4 counts |
| S1 | 33 | M | Heterosexual | | 2005 | ART –ve | 364 |
| S2 | 37 | M | Heterosexual | | 2006 | ART –ve | NA |
| S3 | 35 | F | Heterosexual | | 2004 | ART +ve | 253 |
| S4 | 23 | F | Heterosexual | | 2007 | ART –ve | NA |
| S5 | 29 | M | Heterosexual | | 2008 | ART +ve | 111 |
| S6 | 36 | M | Heterosexual | | 2008 | ART –ve | 345 |
| VT1 | 24 | F | Heterosexual | | 2007 | ART +ve | 152 |
| VT2 | 4 | M | Vertical | | 2007 | ART +ve | 727 |
| VT3 | 30 | F | Heterosexual | | 2008 | ART –ve | 233 |
| VT4 | 8 | M | Vertical | | 2007 | ART +ve | 804 |
| VT5 | 38 | F | Heterosexual | | 2006 | ART –ve | 96 |
| VT6 | 6 | M | Vertical | | 2006 | ART –ve | 1048 |
| VTD8 | 27 | F | Heterosexual | | 2006 | ART –ve | 475 |
| VTE8 | 5 | F | Vertical | | 2006 | ART +ve | 870 |
| D1 | 30 | F | Heterosexual | | 2008 | ART –ve | 419 |
| E1 | 9 | M | Vertical | | 2008 | ART +ve | NA |
| D2 | 30 | F | Heterosexual | | 2008 | ART +ve | 403 |
| E2 | 6 | M | Vertical | | 2008 | ART –ve | 972 |
| D58 | 28 | F | Heterosexual | | 2008 | ART +ve | 257 |
| E58 | 9 | M | Vertical | | 2008 | ART +ve | 680 |
| D60 | 33 | F | Heterosexual | | 2008 | ART +ve | 336 |
| E60 | 9 | M | Vertical | | 2008 | ART –ve | 834 |
| D62 | 30 | F | Heterosexual | | 2008 | ART +ve | 376 |
| E62 | 6 | M | Vertical | | 2008 | ART +ve | 676 |
| D63 | 30 | F | Heterosexual | | 2008 | ART –ve | 212 |
| E63 | 10 | M | Vertical | | 2008 | ART +ve | 458 |
| D64 | 35 | F | Heterosexual | | 2008 | ART +ve | 403 |
| E64 | 7 | M | Vertical | | 2008 | ART +ve | 572 |
| A4 | 30 | M | Heterosexual | | 2004 | ART –ve | 351 |
| A5 | 28 | F | Heterosexual | | 2008 | ART –ve | 972 |
| A6 | 28 | F | Heterosexual | | 2006 | ART –ve | 1046 |
| A7 | 35 | M | Heterosexual | | 2008 | ART –ve | 519 |
| A8 | 39 | M | Heterosexual | | 2006 | ART –ve | 447 |
| A9 | 35 | M | Heterosexual | | 2006 | ART –ve | 440 |
| N11 | 32 | M | Heterosexual | | 2010 | ART +ve | 140 |
| N12 | 27 | F | Heterosexual | | 2010 | ART +ve | 226 |
| N13 | 39 | F | Heterosexual | | 2010 | ART +ve | 104 |
| N14 | 40 | M | Heterosexual | | 2010 | ART +ve | 198 |
| N15 | 50 | M | Heterosexual | | 2010 | ART +ve | 74 |
| N16 | 30 | M | Heterosexual | | 2010 | ART +ve | 256 |
| N17 | 40 | M | Heterosexual | | 2010 | ART +ve | 114 |
| N45 | 30 | M | Heterosexual | | 2010 | ART –ve | 153 |
| N46 | 25 | M | Heterosexual | | 2010 | ART –ve | 125 |
| N47 | 35 | F | Heterosexual | | 2010 | ART –ve | 339 |
| N48 | 24 | F | Heterosexual | | 2010 | ART –ve | 600 |
| N49 | 32 | M | Heterosexual | | 2010 | ART –ve | 717 |
| N50 | 32 | F | Heterosexual | | 2009 | ART –ve | 203 |
| N51 | 25 | M | Heterosexual | | 2010 | ART –ve | NA |
| N52 | 24 | F | Heterosexual | | 2010 | ART –ve | 165 |
| N53 | 30 | M | Heterosexual | | 2010 | ART –ve | 64 |
| N54 | 35 | F | Heterosexual | | 2009 | ART –ve | NA |
| N55 | 36 | M | Heterosexual | | 2010 | ART –ve | 39 |
| N56 | 30 | M | Heterosexual | | 2009 | ART –ve | 110 |
| N57 | 27 | F | Heterosexual | | 2009 | ART –ve | NA |
| N 58 | 45 | F | Heterosexual | | 2009 | ART –ve | 156 |
| N59 | 45 | F | Heterosexual | | 2010 | ART –ve | 56 |
| N60 | 37 | M | Heterosexual | | 2008 | ART –ve | 93 |
| N61 | 24 | M | Heterosexual | | 2008 | ART –ve | NA |
| N62 | 35 | M | Heterosexual | | 2010 | ART –ve | 119 |
| N63 | 30 | M | Heterosexual | | 2010 | ART –ve | 159 |
| N64 | 42 | F | Heterosexual | | 2010 | ART –ve | 146 |
| N65 | 32 | M | Heterosexual | | 2010 | ART –ve | NA |
| N66 | 36 | M | Heterosexual | | 2009 | ART –ve | 591 |
| N67 | 35 | M | Heterosexual | | 2010 | ART –ve | NA |
| N68 | 25 | F | Homosexual | | 2009 | ART +ve | 80 |
| N69 | 25 | F | Heterosexual | | 2007 | ART –ve | 433 |
| N70 | 22 | M | Heterosexual | | 2008 | ART –ve | 78 |
| N71 | 32 | F | Heterosexual | | 2007 | ART +ve | 139 |
| N72 | 27 | F | Homosexual | | 2008 | ART +ve | 131 |
| N73 | 39 | M | Heterosexual | | 2007 | ART +ve | 218 |
| N74 | 40 | M | Heterosexual | | 2008 | ART +ve | 198 |
| N75 | 50 | M | Heterosexual | | 2010 | ART +ve | 74 |
| N76 | 30 | M | Heterosexual | | 2010 | ART –ve | 304 |
| N77 | 40 | M | Heterosexual | | 2010 | ART –ve | 63 |
| N78 | 30 | F | Heterosexual | | 2010 | ART –ve | 358 |
| N79 | 25 | F | Heterosexual | | 2007 | ART +ve | 55 |
| N80 | 34 | M | Heterosexual | | 2009 | ART +ve | 83 |
| N81 | 21 | F | | Heterosexual | 2009 | ART +ve | 176 |
| N82 | 32 | F | Heterosexual | | 2008 | ART –ve | 774 |
| N83 | 50 | F | Heterosexual | | 2009 | ART –ve | 225 |
| N84 | 40 | F | Heterosexual | | 2007 | ART +ve | 265 |
| N85 | 51 | M | Heterosexual | | 2007 | ART +ve | 85 |
| N86 | 32 | M | Heterosexual | | 2007 | ART +ve | 185 |
| N87 | 41 | F | Heterosexual | | 2007 | ART +ve | 114 |
| N88 | 48 | M | Heterosexual | | 2008 | ART –ve | 451 |
| N89 | 13 | M | Vertical | | 2008 | ART –ve | 422 |
| N90 | 32 | M | Heterosexual | | 2009 | ART +ve | 111 |
| N91 | 04 | M | Heterosexual | | 2009 | ART –ve | 301 |
| N92 | 48 | M | Heterosexual | | 2008 | ART +ve | 103 |
| N93 | 19 | F | Heterosexual | | 2008 | ART –ve | 116 |
| N94 | 06 | F | Vertical | | 2009 | ART –ve | 227 |
| N95 | 08 | M | Vertical | | 2009 | ART –ve | 547 |
| N96 | 38 | M | Heterosexual | | 2009 | ART +ve | 90 |
| N97 | 35 | F | Heterosexual | | 2009 | ART –ve | 442 |
| N98 | 03 | F | Vertical | | 2010 | ART –ve | 429 |
| N99 | 25 | M | Heterosexual | | 2009 | ART –ve | 317 |
| N100 | 29 | F | Heterosexual | | 2005 | ART +ve | 114 |
| N101 | 48 | M | Heterosexual | | 2005 | ART +ve | 252 |
| N102 | 34 | F | Heterosexual | | 2008 | ART –ve | 405 |
| N103 | 35 | M | Heterosexual | | 2009 | ART –ve | 49 |
| N104 | 24 | F | Heterosexual | | 2007 | ART –ve | 344 |
| N105 | 26 | M | Heterosexual | | 2007 | ART –ve | 114 |
| N106 | 33 | M | Heterosexual | | 2007 | ART –ve | 171 |
| N107 | 35 | M | Heterosexual | | 2005 | ART –ve | 227 |
| N108 | 30 | M | Heterosexual | | 2006 | ART +ve | 197 |
| N109 | 27 | M | Heterosexual | | 2006 | ART –ve | NA |
| N110 | 35 | F | Heterosexual | | 2009 | ART –ve | 265 |
| N111 | 58 | M | Heterosexual | | 2009 | ART –ve | 88 |
| N112 | 54 | F | Heterosexual | | 2010 | ART –ve | 44 |
| N113 | 43 | M | Heterosexual | | 2005 | ART +ve | 182 |
| N114 | 24 | F | Heterosexual | | 2005 | ART +ve | 345 |
| N115 | 44 | M | Heterosexual | | 2004 | ART +ve | 385 |
| N116 | 31 | F | Heterosexual | | 2005 | ART +ve | 246 |
| N117 | 41 | F | Heterosexual | | 2004 | ART +ve | 176 |
| N118 | 28 | F | Heterosexual | | 2004 | ART +ve | 215 |
| N119 | 54 | M | Heterosexual | | 2007 | ART +ve | 209 |
| N120 | 22 | M | Heterosexual | | 2005 | ART –ve | 481 |
| N121 | 35 | M | Heterosexual | | 2008 | ART +ve | 211 |
| N122 | 14 | F | Heterosexual | | 2004 | ART –ve | 117 |
| N123 | 44 | M | Heterosexual | | 2008 | ART +ve | 98 |
| NA (Not Available); ART –ve (naive); ART +ve (positive);  Letter D denotes mother and E denotes child | | | | | | | |
